# Awareness of Amalgam and Safe Removal Techniques Among Dentists in Turkey: A Cross-Sectional Study

**DOI:** 10.3290/j.ohpd.c_2416

**Published:** 2025-12-12

**Authors:** Cansu Yikici Çöl, Sema Yazici Akbiyik, Feridun Hürmüzlü

**Affiliations:** a Cansu Yikici Çöl Specialist in Restorative Dentistry, Panorama Ankara Oral and Dental Health Clinic, Çankaya, Ankara, Turkey. Writing – review and editing, writing – original draft, methodology, investigation, and data curation.; b Sema Yazici Akbiyik Assistant Professor, Department of Restorative Dentistry, Faculty of Dentistry, Lokman Hekim University, Çankaya, Ankara, Turkey. Writing – review and editing, validation, methodology, formal analysis, data curation, and conceptualisation.; c Feridun Hürmüzlü Professor Doctor, Department of Restorative Dentistry, Faculty of Dentistry, Lokman Hekim University, Çankaya, Ankara, Turkey. Writing – review and editing, validation, supervision, project administration, data curation, and conceptualisation.

**Keywords:** amalgam, awareness, human health, mercury, SMART, removal

## Abstract

**Purpose:**

Mercury toxicity from amalgam restorations is discussed as both a physician–patient issue and an environmental concern. This cross-sectional descriptive study aimed to evaluate the awareness of dentists in Turkey regarding safe amalgam removal protocols.

**Methods and Materials:**

A 42-question online survey addressing sociodemographic characteristics, educational background, and knowledge of amalgam, removal procedures and their alternatives was conducted between May and September 2024, reaching a sample size of 269 participants. Data were analysed using SPSS version 25.0 for Windows, and a P-value <0.05 was considered statistically significant.

**Results:**

Participants reported a 97.2% rate of undergraduate amalgam training, including both theoretical and clinical components; however, only 23.6% were currently using amalgam in practice. Additionally, 39.7% stated their knowledge about safe removal was insufficient or uncertain, and just 45.6% expressed concern about mercury’s environmental impact.

**Conclusion:**

Governments and professional organisations should actively promote environmentally responsible practices through strategies focused on education, awareness, and sustainability to protect public health and natural resources.

Dental amalgam, used for over 150 years, is valued for its durability, low cost, and ease of use in challenging clinical situations. However, its poor aesthetics, non-conservative cavity preparation, and concerns regarding mercury content have limited its appeal. Adhesive restorative systems have largely replaced amalgam due to better aesthetics, biocompatibility, and comparable clinical outcomes, though amalgam may still be preferred in high-caries-risk patients because of its cost-effectiveness and reliability.^22^

Debates over dental amalgam use have intensified, mainly due to its approximately 50% mercury content.^9^ Mercury exposure occurs mostly during placement and removal, with minimal emissions once restorations are set. Inhalation during replacement using rotary instruments constitutes the most significant occupational and patient risk,^35^ while amalgam waste disposal raises additional environmental concerns.^31^

At the international level, the United Nations Environment Programme (UNEP) reported that dental amalgam accounts for approximately 21% of global mercury use in products.^12^ The World Health Organization (WHO, October 2024) has classified mercury as a major public health concern,^36^ and the Minamata Convention on Mercury (2013) emphasised the need to mitigate environmental and health hazards associated with mercury pollution.^34^ In line with these efforts, the European Union introduced Regulation (EU) 2017/852, prohibiting amalgam use in children under 15 years of age and in pregnant or breastfeeding women as part of a broader phase-down strategy.^10^

Despite these initiatives, scientific debate persists. In 2015, the Scientific Committee on Health and Environmental Risks (SCHER) concluded that a definitive assessment of the environmental and indirect health impacts of dental amalgam mercury was not yet feasible, emphasising the need for further evidence.^27^ Nevertheless, when amalgam restorations must be replaced due to secondary caries or fracture, specific clinical protocols have been developed to minimise mercury vapour inhalation and soft tissue exposure, including rubber dam isolation, high-volume suction, and copious water cooling.^7^

In this study, safe amalgam removal refers to adherence to internationally recommended mercury hygiene practices, such as the use of rubber dam isolation, high-volume suction, water cooling, and appropriate personal protective equipment during amalgam removal procedures. Knowledge about safe removal was defined as participants’ awareness of and familiarity with these procedures.

While international organisations such as the WHO, the European Union, and the Minamata Convention have emphasised the reduction of mercury use and the importance of safe removal protocols, there is limited evidence on how these global policies translate into national-level awareness and practices. In Turkey, where amalgam is still part of undergraduate training and continues to be used in clinical practice, understanding dentists’ knowledge and attitudes toward safe removal is critical. This study aimed to fill this gap by providing country-specific data that can both inform national policy development and contribute to the broader international discussion on environmentally responsible dental practices.

The aim of this cross-sectional descriptive study was to evaluate dentists’ perspectives in Turkey on dental amalgam restorations, their knowledge of safe amalgam removal protocols, and their awareness of potential alternatives. The study was designed to raise awareness, improve knowledge levels among practitioners, and draw attention to the environmental and public health impacts of amalgam use.

The null hypothesis of this study was that there would be no statistically significant association between dentists’ sociodemographic or professional characteristics (such as years of experience, field of specialisation, or workplace setting) and their knowledge, attitudes, or practices regarding dental amalgam use, safe amalgam removal protocols (including the SMART technique), or environmental concerns related to mercury exposure.

## METHODS AND MATERIALS

This study was initiated with the approval of the Lokman Hekim University Scientific Research Ethics Committee (Decision No: 2024/125). The present study was conducted in compliance with the ethical standards of the Declaration of Helsinki and in accordance with the Strengthening the Reporting of Observational Studies in Epidemiology (STROBE) guidelines.

This study included dentists (general practitioners, specialists, PhD students, and faculty members) who were actively practising in Turkey and voluntarily agreed to participate by providing informed consent. Dentists who submitted incomplete responses, provided duplicate entries, or were not currently practising in Turkey were excluded. These criteria were applied consistently to ensure the validity and reliability of the study sample.

The sample size was calculated using the Raosoft Web Survey Software (http://www.raosoft.com/samplesize.html) with a 90% confidence interval and a 5% margin of error. Based on a population of 39,851 (according to 2021 statistics from the Turkish Statistical Institute regarding the number of dentists in Turkey), it was determined that a minimum of 269 participants were required. In order to address the possibility of incomplete or invalid data and to strengthen the reliability of the study outcomes, a total of 318 participants were ultimately included in the analysis. Data collection took place between May and September 2024. Inclusion criteria were dentists

All variables of interest were assessed using a standardised self-administered online questionnaire developed following an extensive literature review.^7,29,33^ The survey included 42 questions covering sociodemographic characteristics, educational background, knowledge and attitudes towards amalgam, awareness of safe amalgam removal procedures, and access to alternatives. Content validity was assessed by 10 experts using a four-point relevance scale (1 = not relevant, 4 = highly relevant). The item-level CVI (I–CVI) ranged from 0.85 to 1.00, and the scale-level CVI (S-CVI/Ave) was 0.94, indicating excellent content validity. Based on their feedback, minor revisions were implemented to enhance comprehensibility and appropriateness. As all participants completed the same questionnaire under the same conditions, the methods of assessment were directly comparable across groups. All responses were self-reported. The survey link to the created form was shared between May 2024 and September 2024 on various social platforms frequented by dentists and was also sent via email to all dentists registered with the Turkish Dental Association (TDA). A reminder email was sent 15 to 30 days later. The terms of use and privacy policy of the survey platform used during the study were fully adhered to. Since there were no identifying questions in the survey, participant confidentiality was strictly maintained.

The primary outcomes of the study were dentists’ knowledge, attitudes, and practices regarding dental amalgam use and safe amalgam removal procedures. The secondary outcomes included the associations between these outcomes and participants’ sociodemographic or professional characteristics (such as age, gender, years of professional experience, specialisation, and workplace setting). Additionally, secondary analyses explored awareness of international regulations (eg, the Minamata Convention), knowledge of alternative restorative materials, and reported environmental concerns. Exposures and predictors included sociodemographic characteristics (age, gender), educational background, years of professional experience, and workplace setting. Potential confounders considered were professional position (general dentist, specialist, faculty member) and field of expertise, as these factors may influence both awareness and clinical practices. Effect modification was explored by specialisation, given its potential to alter responses concerning amalgam safety and removal protocols. As this was a questionnaire-based cross-sectional study, no diagnostic criteria were applicable.

Several efforts were undertaken to minimise potential sources of bias. To reduce selection bias, the survey link was distributed through multiple channels, including the TDA registry and professional social media platforms, to maximise reach. Information bias was limited by ensuring anonymity of responses, which minimised social desirability effects. The survey platform incorporated security measures preventing multiple submissions from the same participant. In addition, the questionnaire was pilot-tested and revised for clarity, thereby reducing the risk of misinterpretation and recall bias. Nonetheless, as with all self-reported data, recall and reporting biases cannot be fully excluded.

### Statistical Analysis

Quantitative variables were primarily treated as categorical in the analysis. Years of professional experience were grouped into four categories (0–1, 2–5, 6–10, and ≥11 years) to allow for meaningful comparisons across different career stages. Educational level (undergraduate, specialist, PhD student, faculty member) and workplace setting (private practice, Ministry of Health institution, university hospital) were similarly categorised. The data obtained in the study were analysed using the SPSS (Statistical Package for Social Sciences) for Windows, version 25.0 software. Descriptive statistical methods (number, percentage) were used to evaluate the data. Relationships between categorical variables were examined using Chi-square analysis. Relationships between categorical variables were examined using Chi-square analysis. A value of P <0.05 was considered statistically significant. In cases where statistically significant associations were observed, effect size was calculated using Cramer’s V to assess the strength of the relationship (very weak <0.1, weak = 0.1, moderate = 0.3, strong = 0.5).

## RESULTS

The survey consisted of a total of 42 questions. The first 8 questions were aimed at determining the dentists’ sociodemographic characteristics and educational background. Questions 9 to 14 inquire about undergraduate education and familiarity with the amalgam debates. Questions 15 to 22 assess knowledge regarding mercury content and the safety of amalgam. Questions 23 to 42 investigate topics such as the replacement of amalgam fillings, alternatives to amalgam, and knowledge about safe amalgam removal procedures.

### Sociodemographic Information and Educational Status

The distribution of the participants based on their sociodemographic characteristics is summarised in Table 1.

**Table 1:** Sociodemographic characteristics of the participants

Variables	n	%
Gender	Female	172	54.1
Male	146	45.9
Working time in dentistry	0–1 year	38	11.9
2–5 years	55	17.3
6–10 years	62	19.5
+11 years	163	51.3
Level of education	5 years of dentistry education	187	58.8
PhD student	38	11.9
Specialist dentist	93	29.2
Position in the institution	Dentist	221	69.5
Specialist dentist	57	17.9
Faculty member	40	12.6
Working time as a specialist dentist	0–1 year	9	2.8
2–5 years	37	11.6
6–10 years	19	6.0
+11 years	32	10.1
Total	97	30.5
Field of expertise	Oral, dental and maxillofacial radiology	6	1.9
Oral and maxillofacial surgery	11	3.5
Endodontics	9	2.8
Orthodontics	9	2.8
Pedodontics	9	2.8
Prosthetic dentistry	20	6.3
Restorative dentistry	25	7.9
Periodontology	8	2.5
Total	97	30.5
Institution	Private clinic/Polyclinic	204	64.2
Institutions affiliated with the Ministry of Health	32	10.1
University hospital	82	25.8
Total	318	100.0


### Undergraduate Education and Familiarity with Amalgam Debates

Amalgam was included in the undergraduate curriculum (including both theoretical and clinical components) for 97.2% (n = 309) of participants; 92.5% (n = 294) practised on models and 78% (n = 248) on patients. Currently, 23.6% (n = 75) still use it in practice. Regarding awareness, 84.6% (n = 269) were familiar with debates, mainly informed through undergraduate education (50.6%, n = 161), questions from patients (49.4%, n = 157), conferences (45.6%, n = 145), media (37.1%, n = 118), colleagues (48.7%, n = 155), and other sources (8.5%, n = 27).

### Knowledge Level Regarding Mercury Content and Amalgam Safety

When asked about mercury content, 35.8% (n = 114) rated their knowledge as good, 56.3% (n = 179) as moderate, and 7.9% (n = 25) as poor. Regarding safety, 13.5% (n = 43) said patients considered amalgam safe, 56% (n = 178) unsafe, and 30.5% (n = 97) were unsure. Additionally, 87.4% (n=278) reported patient concern about the amalgam’s colour.

When asked about mercury toxicity, 71.4% (n = 227) correctly identified vapour as the most toxic form. Similarly, 66% (n = 210) correctly answered that mercury is most rapidly absorbed through the lungs. While 37.1% (n = 118) viewed amalgam as safe, 40.3% (n = 128) did not, and 22.6% (n = 72) were unsure. Opinions on banning amalgam were divided: 34.9% (n = 111) supported a ban, 35.2% (n = 112) opposed it, and 29.9% (n = 95) were unsure. Only 24.2% (n = 77) had heard of the Minamata Convention.

### Replacement of Amalgam Fillings, Amalgam Alternatives, Safe Amalgam Removal Protocols

Most participants (92.5%, n = 294) believed amalgam should only be replaced if damaged, while 4.7% (n = 15) would do so upon patient request and 2.8% (n = 9) favoured replacement regardless. Regarding alternatives to amalgam, 56.9% (n = 181) rated their knowledge as good, and 84.6% (n = 269) had access to such materials. About 48.4% (n = 154) were familiar with the Safe Mercury Amalgam Removal Technique (SMART) protocol. About 60.4% (n = 192) felt adequately informed about safe amalgam removal, while 34.3% (n = 109) supported doing it only in controlled environments; 39.6% (n = 126) disagreed, and 26.1% (n = 83) were unsure.

Amalgam removal had been performed by 95.3% (n = 303) of participants. Among them, 38.7% (n = 123) used a rubber dam, 18.2% (n = 58) used detox agents with cotton rolls, and 27.4% (n = 87) applied protective drapes. Protective clothing was used by 78.9% (n = 251), and 7.9% (n = 25) provided nasal oxygen. Technically, 77% (n = 245) used new burs, 80.8% (n = 257) used high-volume suction, 96.9% (n = 308) used water cooling, 95.9% (n = 306) removed the amalgam in large chunks, and 97.2% (n = 309) rinsed thoroughly afterwards. Only 7.2% (n = 23) had a certified amalgam separator installed, 57.5% (n = 183) reported not having one, and 35.2% (n = 112) were unsure whether one was installed. About 16.7% (n = 53) recommended vitamin C around amalgam removal. While 45.6% (n = 145) were concerned about mercury’s environmental impact, 29.9% (n = 95) were not, and 24.5% (n = 78) lacked knowledge. Responses on various topics, such as amalgam safety, alternatives, and removal, were also compared by years in practice, position, and institution.

These results are shown in Tables 2, 3, and 4. Figure 1 summarises safe amalgam removal protocols by speciality.

**Table 2:** Some important questions compared to your professional year

	2: How many years have you been a dentist? (N = 318)
0–1 year	2–5 year	6–10 year	+ 11 years
**11. Have you ever applied amalgam fillings to patients under clinical conditions during your undergraduate education? (Who said yes)**	31.60%	47.30%	87.10%	95.70%
**12. Do you use amalgam fillings in your clinical practice? (Who said yes)**	10.50%	16.40%	24.20%	28.80%
**13. Are you familiar with the discussions about amalgam? (Who said yes)**	71.10%	78.20%	80.60%	91.40%
**15. What is your level of knowledge about the ‘mercury’ contained in amalgam fillings? (Who said poor)**	18.40%	7.30%	3.20%	7.40%
**18. Which is the most toxic form of mercury? (Incorrect answers)**	47.40%	52.70%	21.00%	19.00%
**19. Mercury is absorbed fastest in ………. (Choose the most appropriate option for you in the blank) (Incorrect answers)**	34.20%	47.30%	25.80%	32.50%
**21. What do you think about the contradictions regarding the amalgam ban? (Those who say it should be banned)**	42.10%	47.30%	30.60%	30.70%
**22. Have you heard of the Minimata Convention? (Who said yes)**	18.40%	16.40%	29.00%	26.40%
**23. Which of the following is more appropriate for you regarding the need to change an amalgam filling in the mouth? (Who say ‘it should be changed regardless of whether there is a problem or not’)**	2.60%	3.60%	1.60%	3.10%
**25. Do you have access to alternative materials to amalgam? (Those who said no)**	10.50%	9.10%	6.50%	2.50%
**26. Do you know the SMART protocol? (Those who said no)**	44.70%	47.30%	41.90%	58.30%
**29. Have you ever removed an amalgam filling in your career? (Who said yes)**	86.80%	96.40%	93.50%	97.50%
**42. Are you bothered by the environmental issues of mercury in the dentist’s office? (No)**	28.90%	23.60%	22.60%	35.00%


Dentists with more than 11 years of professional experience demonstrated a higher accuracy (81%) in identifying mercury vapour as the most toxic form compared to those with 0–1 years (52.6%). This association was statistically significant, χ² (3,N = 318) = 31.32, P <0.05, with a moderate effect size (Cramer’s V = 0.31). Specialisation also mattered, as 100% of specialists in radiology, endodontics, and paediatric dentistry answered correctly. The association was statistically significant χ^2^(7, N = 97) = 32.64, P <0.001, with a strong effect size (Cramer’s V = 0.58).

The relationship between speciality area and the perceived necessity of replacing existing amalgam restorations was statistically significant (χ^2^ (14, N = 97) = 27.671, P = 0.016), with the effect size as moderate (Cramer’s V = 0.378); all specialists in radiology, paediatric dentistry, prosthodontics, and restorative dentistry supported replacement only if clinical issues were present. Access to amalgam alternatives was also tied to workplace type (χ^2^ (4, N = 318) = 11.118, P = 0.025, Cramer’s V = 0.132), with 88.2% of those in private settings reporting access.

Specialisation was significantly linked to awareness of the SMART protocol (χ^2^(7, N = 97) = 25.873, P = 0.001,Cramer’s V = 0.516), with 88.9% of paediatric dentistry specialists familiar with it. The same group also showed high use of rubber dams (88.9%) and reported amalgam removal experience across all paediatric, prosthodontic, and restorative specialists (χ^2^(7, N = 97) = 21.298, P = 0.003, Cramer’s V = 0.468). No significant relationship was found between years of practice and environmental concerns (χ^2^ (6, N = 318) = 7.819, P = 0.252, Cramer’s V = 0.111).

Among restorative dentistry specialists, 12% (n = 3) used detox agents with cotton rolls, 28% (n = 7) applied protective drapes, and 76% (n = 19) used protective clothing. Only 4% (n = 1) provided nasal oxygen. Technically, 76% (n = 19) used new burs, 64% (n = 16) used high-volume suction, and all removed amalgam in chunks under water cooling; 96% (n = 24) rinsed afterwards. Only 8% (n = 2) had certified amalgam separators, and 20% (n = 5) recommended vitamin C.

## DISCUSSION

The null hypothesis was partially rejected, as significant associations were found between occupational characteristics (eg, specialisation and years of experience) and knowledge of mercury toxicity or familiarity with the SMART protocol. These findings highlight the influence of occupational background on amalgam awareness and safe amalgam removal protocols.

According to the results of the study, the use of amalgam during undergraduate education and the preference for amalgam fillings in clinical practice have significantly declined over the years in Türkiye. For this reason, it was observed that more experienced dentists (11+ years) were more familiar with the debates surrounding amalgam, although they tended to demonstrate a lower level of specific knowledge about mercury itself.

Although the majority of participants reported receiving undergraduate education on amalgam, this training appears to have emphasised technical aspects of restorative procedures rather than the toxicological, environmental, and occupational health implications of mercury. Furthermore, the declining use of amalgam in daily practice may have led to reduced exposure to relevant protocols, resulting in knowledge attrition over time. This pattern is consistent with previous reports showing limited awareness of amalgam safety and disposal practices among both students and practitioners.^9,31^ A similar study^31^ conducted in Greece reported that dental students no longer receive education or training in amalgam use during their undergraduate studies. While younger dentists may lack experience with amalgam restorations, this study found that amalgam remains part of Türkiye’s dental curriculum (97.2%, n = 309). As the first study in Turkey on dentists’ knowledge of safe amalgam removal, it highlights the need to improve awareness of the SMART protocol, mercury-related risks, environmental concerns, and alternative materials.

A recent cross-sectional study^3^ from Iraq reported comparable findings, indicating that although many dentists had received formal education on amalgam, their awareness regarding its mercury content and environmental implications remained limited. In that study, only 22.69% of Iraqi dentists preferred using amalgam, while the majority avoided it mainly for aesthetic and safety concerns, with mercury content cited as a major reason for restriction. Similarly, more than half of the surveyed patients were unaware of the type or composition of their restorations, and aesthetic preferences largely influenced their choices. These findings parallel the present study’s results, which also demonstrate a gap between formal education and practical awareness of mercury safety among dental professionals. Together, these results underscore the need for more comprehensive education and policy initiatives in dental curricula and continuing professional development programmes to strengthen understanding of the environmental and occupational health aspects of amalgam use.

Concerns about mercury toxicity from dental amalgam date back to 1843.^13^ Although studies have examined its long-term effects on development, immune, renal, and neurological functions, current evidence does not indicate systemic health risks for the general population^4,5,6,8,19,21,30^ but its global use is declining due to its poor aesthetics.^23^ Major health authorities, including the WHO, FDA, FDI, and SCENIHR, regard dental amalgam as safe, with risks mainly limited to local effects, even for vulnerable groups like children and pregnant women.^2^

Awareness of mercury’s neurotoxic potential has led to the development of stringent safety protocols for amalgam removal. Professional organisations such as the International Academy of Oral Medicine and Toxicology (IAOMT) and the International Academy of Biological Dentistry and Medicine (IABDM) have introduced evidence-based frameworks, including the SMART and the PROTECT Protocol, to support safe clinical practice. These recommendations highlight strategies to limit mercury exposure, such as the use of high-volume evacuation systems, rubber dams, provision of oxygen via nasal cannula, and comprehensive protective garments. Further safeguards involve applying copious cold-water irrigation to reduce vapourisation, sectioning restorations to minimise particle release, and maintaining effective ventilation within the operatory to protect both patients and dental staff.^32^

To the question ‘Do you know the SMART protocol?’, 51.6% (n = 164) of all participants (n = 318) answered ‘no’. According to the results of this cross-sectional study, we can say that knowledge about SMART is limited. Beyond chairside protections, environmental policies mandate separators to curb mercury release (EU Regulation 2017/852; US EPA 40 CFR Part 441), reinforcing the clinical and public health rationale for broader adoption of SMART-consistent practices.^1^ Thus, dentists must be well-informed about safe amalgam removal protocols and associated risks.

This study aimed to investigate dentists’ familiarity with dental amalgam and its mercury content, their knowledge and undergraduate education, views on the replacement of amalgam fillings, awareness of amalgam alternatives, and understanding of safe amalgam removal protocols.

It was observed that 84.6% (n = 269) of the participants were familiar with the ongoing discussions about amalgam, despite the fact that 97.2% (n = 309) had received training on amalgam during their undergraduate education. Currently, 23.6% (n = 75) of the participants continue to use amalgam in their clinical practice. On the other hand, 62.9% (n = 200) responded ‘no’ or ‘not sure’ to the question, ‘Do you think amalgam is a safe restorative material?’, while 64.2% (n = 204) rated their knowledge of mercury content in amalgam as moderate or poor. In addition, 29.9% (n = 95) reported being unsure about a potential ban on amalgam.

These findings reflect a degree of uncertainty and inconsistency within the current dental education system and clinical practices in Turkey. This situation should be considered a significant indicator for the need to update educational curricula and reevaluate the topic of dental amalgam within professional training and public health policy.

When asked ’What is your patients’ opinion about amalgam safety?’, 56% (n = 178) responded ’not safe’, and 30.5% (n = 97) responded ’not sure’. This represents an increase compared to a previous study,^2^ in which 22.4% of participants stated that amalgam was ‘not safe’ for both the practitioner and the patient, and 16.8% were ‘not sure’. Similar to a previous study,^2^ this study did not find a significant difference (P = 0.472 > 0.05, Cramer’s V ≈ 0.07) in the opinions of general dentists, specialist dentists, and academicians regarding a potential ban on amalgam. While 66.6% (n = 660) of general dentists in the previous study stated that amalgam should not be banned, and 26.1% (n = 259) believed it should be banned, in the current study, 33% (n = 73) expressed that it should not be banned, whereas 38% (n = 84) supported a ban. This indicates a noticeable shift toward increased support for banning amalgam over time.

Current literature supports removing amalgam only when clinically necessary. In line with this, 92.5% (n = 294) of participants agreed, while 2.8% (n = 9) supported removal regardless of clinical need.

The safety of dental amalgam remains debated due to mercury’s known toxicity.^35^ Concerns over its potential health effects, including immunologic disorders, are driving the push for removal. Since mercury particles and vapours are released during removal, minimising exposure is critical for both patients and dental professionals.^14,24,17^ In this study, 51.6% (n = 164) of participants responded ‘no’ to the question, ‘Do you know the SMART protocol?’ Additionally, when asked, ‘What is your level of knowledge about the precautions needed during safe amalgam removal?’, 39.7% (n = 126) rated their knowledge as insufficient or were unsure. This relatively high rate may be interpreted as a sign that the current dental education system requires reassessment, or as a reminder of the need to encourage practitioners to stay updated with current literature.

Since 1 January 2018, the EU Mercury Regulation (2017/852) has banned mercury imports and restricted amalgam use, requiring clinics to use certified separators. It also regulates mercury in industry and waste management.^11^ Similarly, the Minamata Convention promotes a global phase-down of dental amalgam due to environmental concerns from mercury emissions and improper disposal.^2,20^ In the current study, 75.8% (n = 241) of the participants reported that they had not heard of the Minamata Convention, and 92.7% (n = 295) either did not have a certified amalgam separator installed in their clinic’s wastewater system or were unaware of its existence.

Among restorative dentistry specialists, 64% (n = 16) did not use a rubber dam during amalgam removal. In contrast, 88.9% (n = 8) of paediatric dentistry specialists reported using one. While 12.0% (n = 3) of restorative specialists reported using chelating agents such as chlorella or activated charcoal, 80.0% (n = 18) did not cover the patient’s face and clothing with protective drapes. Additionally, 24.0% (n = 6) stated that neither the patient nor the dentist used protective clothing, and 96.0% (n = 24) did not administer nasal oxygen during the procedure. Even a readily accessible technique, such as using a fresh bur to facilitate quicker removal of amalgam, was relatively underutilised. Usage rates by specialty were as follows: paediatric dentistry and orthodontics (88.9%, n = 8) > prosthodontics (85%, n = 17) > oral and maxillofacial radiology (83.3%, n = 5) > oral and maxillofacial surgery (81.8%, n = 9) > endodontics (77.8%, n = 7) > restorative dentistry (76%, n = 19) > periodontology (75%, n = 6). Notably, the highest rates of not using high-volume suction were observed among restorative dentistry (36.0%, n = 9) and endodontics (33.3%, n = 3) specialists. In general, most specialities reported attempting amalgam removal under copious water cooling and in large fragments, followed by thorough oral cavity rinsing. The recommendation to use vitamin C supplementation before and after amalgam removal was largely absent, with non-recommendation rates ranging between 66.7% and 100.0% across specialities.

Different restorative materials are required for different clinical situations, and no single material can universally replace dental amalgam in all applications.^28^ In this study, 95.3% (n = 303) of the participants reported having performed amalgam removal at some point during their professional careers. In contrast, only 1.9% (n = 6) rated their knowledge of alternative materials as ‘poor’, while 15.4% (n = 49) indicated either a lack of access to such materials or uncertainty about their availability.

Green dentistry is a sustainable approach to dental care that seeks to minimise the environmental footprint of clinical practices while enhancing patient and public health. According to the Eco-Dentistry Association (EDA), green dentistry encompasses practices aimed at reducing waste and pollution, conserving energy and water, lowering operational costs, integrating advanced technologies, and prioritising health-conscious methods. The association of amalgam with green dentistry is primarily due to its significant environmental impact. Amalgam typically contains 50% mercury, and dental offices generate approximately 3.7 tonnes of mercury waste annually. Improper disposal of amalgam can lead to the release of mercury into the environment, posing substantial ecological risks. The safe disposal of amalgam waste presents several challenges related to the harmful effects of mercury release. To address this, the American Dental Association (ADA) recommends the ‘GRIT’ action module for managing amalgam waste. This module includes the following steps: grey-bagging the waste, recycling it, installing amalgam separators, and educating practitioners. Amalgam waste can be effectively managed using separators like chair-mounted traps and vacuum filters.^18^ Studies also show that mercury and silver can be recovered and recycled from preclinical amalgam waste.^26^

Amalgam waste can be effectively managed using separators like chair-mounted traps and vacuum filters. Studies also show that mercury and silver can be recovered and recycled from preclinical amalgam waste.

Eco-friendly dental approaches – often referred to as green dentistry – offer dental professionals an opportunity to integrate sustainable and responsible practices into daily care. This study highlights that although most dentists in Turkey received education on amalgam use, their awareness regarding mercury content and safe removal procedures remains limited. Strengthening education on environmental sustainability, safe mercury handling, and waste management is therefore essential. Governments, universities, and professional organisations should collaborate to promote environmentally responsible dental practices and ensure the safe disposal of mercury-containing materials. Comprehensive strategies focused on education, awareness, and environmental stewardship are crucial for safeguarding patient and practitioner health, and for protecting vital natural resources such as water,^16^ soil,^15^ and air^25^ for future generations.

The generalisability of these findings should be interpreted with caution. Although the survey included participants from private practices, university hospitals, and public institutions across Turkey, participation was voluntary and limited to dentists with internet access, which may have introduced selection bias. Consequently, the results may overrepresent dentists who are more interested or informed about the topic. Nonetheless, the inclusion of respondents with diverse institutional affiliations, positions, and years of experience supports an acceptable level of external validity. Additionally, categorising continuous variables such as age and years of experience for subgroup comparisons may have reduced the ability to detect subtle dose–response relationships. Future studies should employ continuous or more detailed analytical approaches to explore these associations more precisely and to evaluate the long-term effects of educational and policy interventions promoting green dentistry.

### Acknowledgements

#### Ethics approval and consent to participate declarations

This study was conducted with the approval of the ethics committee number 2024/125 from the Scientific Research Ethics Committee of Lokman Hekim University. This study adhered to the Declaration of Helsinki, and informed consent to participate was obtained from all of the participants in the study.

**Table 3:** Some important questions compared to the position

	5. What is your position in the institution you work for? (N = 318)
**Dentist**	**Specialist dentist**	**Lecturer**
**11. Have you ever applied amalgam fillings to patients under clinical conditions during your undergraduate education? (Who said yes)**	73.80%	87.70%	87.50%
**12. Do you use amalgam fillings in your clinical practice? (Who said yes)**	23.50%	26.30%	20.00%
**13. Are you familiar with the discussions about amalgam? (Who said yes)**	83.30%	91.20%	82.50%
**15. What is your level of knowledge about the ‘mercury’ contained in amalgam fillings? (Who said poor)**	8.60%	5.30%	7.50%
**18. Which is the most toxic form of mercury? (Incorrect answers)**	31.70%	29.80%	10.00%
**19. Mercury is absorbed fastest in ………. (Choose the most appropriate option for you in the blank) (incorrect answers)**	38.00%	28.10%	20.00%
**21. What do you think about the contradictions regarding the amalgam ban? (Those who say it should be banned)**	38.00%	28.10%	27.50%
**22. Have you heard of the Minimata Convention? (Who said yes)**	21.30%	19.30%	47.50%
**23. Which of the following is more appropriate for you regarding the need to change an amalgam filling in the mouth? (Who say ‘it should be changed regardless of whether there is a problem or not’)**	3.20%	3.50%	0.00%
**25. Do you have access to alternative materials to amalgam? (Those who said no)**	6.30%	1.80%	5.00%
**26. Do you know the SMART protocol? (Those who said no)**	55.20%	43.90%	42.50%
**29. Have you ever removed an amalgam filling in your career? (Who said yes)**	96.80%	91.20%	92.50%
**42. Are you bothered by the environmental issues of mercury in the dentist’s office? (No)**	30.30%	36.80%	17.50%


**Table 4:** Some important questions compared to the institution

	8. Which institution do you work at? (N = 318)
Private clinic/polyclinic	Institutions affiliated with the Ministry of Health	University hospital
**11. Have you ever applied amalgam fillings to patients under clinical conditions during your undergraduate education? (Who said yes)**	80.40%	84.40%	69.50%
**12. Do you use amalgam fillings in your clinical practice? (Who said yes)**	17.60%	50.00%	28.00%
**13. Are you familiar with the discussions about amalgam? (Who said yes)**	88.70%	68.80%	80.50%
**15. What is your level of knowledge about the ‘mercury’ contained in amalgam fillings? (Who said poor)**	7.40%	6.30%	9.80%
**18. Which is the most toxic form of mercury? (Incorrect answers)**	28.40%	28.10%	29.30%
**19. Mercury is absorbed fastest in ………. (Choose the most appropriate option for you in the blank) (Incorrect answers)**	34.80%	28.10%	34.10%
**21. What do you think about the contradictions regarding the amalgam ban? (Those who say it should be banned)**	36.30%	21.90%	36.60%
**22. Have you heard of the Minimata Convention? (Who said yes)**	20.10%	25.00%	34.10%
**23. Which of the following is more appropriate for you regarding the need to change an amalgam filling in the mouth? (Who say ‘it should be changed regardless of whether there is a problem or not’)**	3.40%	3.10%	1.20%
**25. Do you have access to alternative materials to amalgam? (Those who said no)**	3.90%	15.60%	4.90%
**26. Do you know the SMART protocol? (Those who said no)**	54.90%	46.90%	45.10%
**29. Have you ever removed an amalgam filling in your career? (Who said yes)**	96.10%	90.60%	95.10%
**42. Are you bothered by the environmental issues of mercury in the dentist’s office? (No)**	37.70%	15.60%	15.90%


**Fig 1:** Safe amalgam removal methods according to areas of expertise.


